# Intermittent heat exposure and thirst in rats

**DOI:** 10.14814/phy2.12767

**Published:** 2016-04-19

**Authors:** Christopher C. Barney, David M. Kuhrt

**Affiliations:** ^1^ Department of BiologyHope CollegeHollandMichigan

**Keywords:** Angiotensin II, evaporative water loss, thermal dehydration, urine output, water intake

## Abstract

Adequate water intake, supporting both cardiovascular function and evaporative cooling, is a critical factor in mitigating the effects of heat waves, which are expected to increase with global warming. However, the regulation of water intake during periods of intermittent heat exposure is not well understood. In this study, the effects of access to water or no access during intermittent heat exposure were assessed using male Sprague‐Dawley rats exposed to 37.5°C for 4 h/day. After 7 days of intermittent heat exposure, reductions in evaporative water loss were observed in all animals and reductions in water intake following heat exposure occurred as the days of heat exposure increased. Rats that were not allowed water during the 7 days of exposure had decreased rehydration levels, however, rats allowed access to water increased water intake during exposure and exhibited higher overall rehydration levels over the same time period. Peripheral administration of angiotensin II, mimicking activation of volemic thirst, or hypertonic saline solution, activating intracellular thirst, did not result in alteration of water intake in rats exposed to heat with access to water compared to control rats. In contrast, rats exposed to heat without access to water had reduced water intake after administration of hypertonic saline and increased water intake after administration of angiotensin II compared to control rats. These experiments demonstrate that thirst responses to intermittent heat exposure are altered by providing water during heat exposure and that intermittent heat exposure without access to water alters drinking responses to both intracellular and extracellular thirst challenges.

## Introduction

Understanding physiological and behavioral responses of animals, including humans, to heat exposure is becoming of more critical importance with global warming and the expectation of an increase in the number and severity of heat waves (Greene et al. 2011; Huang et al. 2011). For example, thermal dehydration, a negative water balance caused by heat exposure, can rapidly lead to heat exhaustion and heat stroke. Activities that increase water losses during heat exposure, such as hypertension and exercise (Barney et al. 1999; Maughan et al. 2015), and factors that affect thirst, such as aging (Miescher and Fortney 1989; Whyte et al. 2004; Johnson 2007), are likely contributing factors to thermal dehydration. In contrast, in men, acclimation to the heat through intermittent exposure has been shown to decrease the risk of thermal dehydration following exercise in the heat by increasing water intake (Greenleaf et al. 1967, 1983; Edholm 1972). A further understanding of the effects of heat acclimation on water intake is needed as we face greater risks of heat stress with global warming.

The mechanisms responsible for heat acclimation are not fully understood. In rats, however, it has been demonstrated that intermittent heat exposure, like chronic heat exposure, results in a reduction in core temperature and metabolic rate, an increase in heat loss capacity, and shifts in core temperature thresholds for activation of heat loss pathways during acute heat exposure (Horowitz and Samueloff 1979; Kuroshima et al. 1982; Horowitz et al. 1983; Epstein et al. 1990; Shido and Nagasaka 1990a,b; Shido et al. 1991a, 1995; Schwimmer et al. 2004). These thermoregulatory changes suggest that intermittent heat exposure in rats might alter evaporative water loss and thus thermal dehydration‐induced thirst. Further understanding of how water access during intermittent heat exposure affects acclimation to hot environments has the potential to lessen the risks of thermal dehydration.

Acute exposure of rats to a hot environment leads to numerous physiological and behavioral responses. Rats initially become more active and skin and core temperatures increase (Lewis et al. 1960; Hainsworth 1967; Gordon 1993). Increased body temperature activates heat loss mechanisms, the first being vasodilation of tail blood vessels to increase tail skin temperature (Rand et al. 1965; Gordon 1993). Extended heat exposure induces the behavior of saliva spreading, which results in evaporative cooling (Hainsworth 1967, 1968; Yanase et al. 1991). A second behavioral response to heat exposure, drinking, occurs if water is available (Hainsworth et al. 1968; Stricker and Hainsworth 1970). Increased water intake during heat exposure improves heat tolerance and survival time by replenishing water lost through saliva spreading and thus reducing dehydration (Stricker and Hainsworth 1970; Epstein et al. 1990; Zurovski et al. 1991). Thermal stimulation and thermal dehydration are the two primary stimuli for increased water intake during heat exposure. An initial, albeit small, increase in water intake during the first hour of heat exposure, before saliva spreading begins and evaporative water loss increases, is due to thermal stimulation (Lund et al. 1969; Grace and Stevenson 1971; Barney and Folkerts 1995). However, most of the heat‐associated water intake is due to the thermal dehydration that occurs as a result of evaporative water loss (Hainsworth et al. 1968; Lund et al. 1969; Stricker and Hainsworth 1970; Grace and Stevenson 1971; Barney and Folkerts 1995).

Less is known about how intermittent heat exposure may alter thermoregulatory and water balance responses to acute heat exposure in rats. Rats exhibited lower activity levels and weight loss on the second day of acute heat exposure (Lewis et al. 1960) and rats repeatedly exposed to a hot environment for 4–5 h/day at the same time each day had reductions in activity, metabolic rate, and core temperature at 24°C (Shido et al. 1989, 1991a, 1995; Shido and Nagasaka 1990a; Sugimoto et al. 1995), but only at that same time of day. Repeated heat exposure did not alter evaporative heat loss at 24°C (Shido and Nagasaka 1990a; Shido et al. 1991b), but did increase the capacity for non‐evaporative heat loss (Shido and Nagasaka 1990a). The effect of intermittent heat exposure on the magnitude of thermally activated evaporative water loss has not yet been reported. In this study, we sought to test whether access to water during repeated intermittent heat exposures would alter water balance as well as whether intermittent heat acclimation would alter the drinking response to cellular and volemic thirst challenges.

## Materials and Methods

### Animals

Male Sprague‐Dawley rats purchased from Harlan Laboratories (Indianapolis, IN) were used for these experiments. The rats were housed singly in plastic cages with hardwood bedding in an animal facility maintained at 22 ± 2°C and illuminated from 6:00 am until 6:00 pm. While in their home cages, the rats were allowed Purina rat chow and tap water ad libitum. The rats were housed for at least 2 weeks before beginning experiments. The experiments were approved by the Hope College Animal Care and Use Committee and followed Hope College's animal care and use guidelines and the *NIH Guide for the Care and Use of Laboratory Animals*.

### Intermittent heat exposure with and without access to water

In the first experiment, rats were divided into two groups of six rats each, a group which had access to water during heat exposure (IH_w_) and a group that did not have access to water during heat exposure (IH_w/o_). The rats were exposed to the heat for 4 h a day for 7 days, beginning at 8:30 am each day. Prior to the beginning of each heat exposure, the rats’ bladders were emptied by gentle suprapubic pressure and the rats were weighed. Rats were then placed in modified (Barney and West 1990) Nalgene metabolism cages without access to food, which were moved into an environmental chamber kept at 37.5 ± 0.5°C. The rats receiving water during the exposure period were provided with a preweighed water bottle containing water at 37.5 ± 0.5°C. After four hours, the cages were removed from the heat and the rats were reweighed and placed in standard Nalgene metabolism cages in a 25 ± 0.5°C environmental chamber. Bottles with 25 ± 0.5°C water were provided to all animals and water intake and urine output were measured at 1, 2, and 3 h of access to water. Food was not provided during this 3‐hour period. Evaporative water loss during the exposure period was estimated by subtracting urine and feces losses and water intake from the change in body weight over the 4‐hour exposure period. Percent rehydration was determined by dividing the total water intake (exposure period and recovery period) by the evaporative and urinary water losses during the exposure period plus the urine loss during the recovery period, and then multiplying by 100%.

### Thirst challenge experiments

Three groups of 12 rats each were used for this study. Thermal dehydration experiments were carried out on the seventh and 47th day of the study, the angiotensin II and hypertonic saline experiments were carried out on the 30th day of the study and the water deprivation experiment was carried out on the 38th and 39th day of the study. The first group (IH_w_) was exposed to 37.5 ± 0.5°C with access to water for 4 h in an environmental chamber beginning at 8:30 am each day (except for the days on which thirst challenge experiments were carried out) for 50 days, while the second group (IH_w/o_) was similarly exposed to heat but was not given access to water during the exposure period. The control group (Con_w_) was treated exactly like the IH_w_ group, but was exposed to a 25 ± 0.5°C environment each day. The rats were housed singly in their home cages during the exposure periods except as noted below. The rats were weighed at the beginning of the experiments, before each experiment, and occasionally at other times.

Starting at 8:30 am on the seventh and 47th day of the study, all rats had their bladders voided and were weighed. The animals were then transferred to the modified Nalgene cages which were placed in an environmental chamber kept at 37.5 ± 0.5°C for 4 h without access to food or water. Following the exposure period, urine output and evaporative water loss were determined as in experiment 1. Water intake, urine output, and percent rehydration were then determined during a 3‐hour period of access to water at 25 ± 0.5°C as in the first experiment.

On the 30th day of the experiment, the rats were not exposed to the heat. Instead, the rats in each group were divided into two subgroups of six rats each. The rats in the first subgroups received 200 *μ*g angiotensin II (Sigma)/kg injected intraperitoneally (i.p.) at 1:00 pm. Thirty minutes later, the rats were placed in Nalgene metabolism cages and given access to water but not food in an environmental chamber at 25 ± 0.5°C. Water intake and urine output were determined after 1, 2, and 3 h. The rats in the second subgroup were lightly anesthetized with methoxyflurane and then injected with hypertonic saline solution (10 mL/kg of 37°C, 0.75 mol/L NaCl, i.p.). The rats were placed in metabolism cages with access to water, but not food 30 min after the injection, and water intake and urine output were determined after 1, 2, and 3 h.

On the 38th day of the experiments, all rats were deprived of water for a 24 h period but not exposed to the heat. All animals were left in their home cages in the animal facility and deprived of water, but not food, beginning at 1:00 pm. At 1:00 pm on the next day, the animals had their bladders voided and were weighed prior to being moved into Nalgene metabolism cages at 25 ± 0.5°C. Animals were provided with water but not food and water intakes and urine outputs of the rats were then determined at 1, 2, and 3 h after the 24 h water deprivation period.

### Statistical analysis

The software package SYSTAT 11 was used for statistical analysis. The data are expressed as means ± SE. Data were analyzed with *t*‐tests, one‐way analysis of variance (ANOVA), one‐way ANOVA with repeated measures, two‐way ANOVA, and two‐way ANOVA with repeated measures. Tukey's test was used for post hoc pairwise comparisons of means. Significance was set at the 95% confidence level.

## Results

### Access to water during intermittent heat exposure increases total water intake and improves rehydration

**Figure 1:**
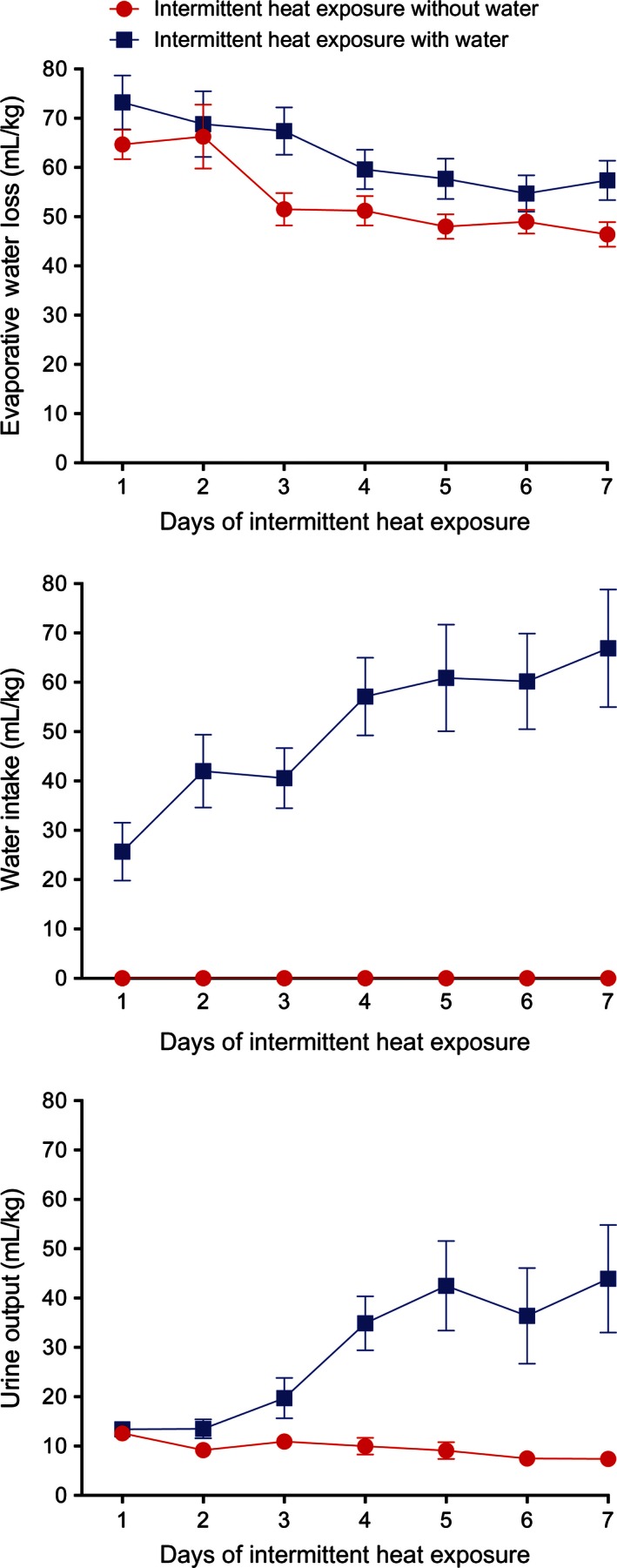
Mean ± SE evaporative water loss (top), water intake (middle), and urine output (bottom) during 4 h of exposure to 37.5°C for seven consecutive days with and without access to water. *N* = 6 per group. There was a significant (*P* < 0.005) change in evaporative water loss over time in both groups with evaporative water loss being significantly (*P* < 0.01) higher in the group with access to water. The group with access to water significantly increased (*P* < 0.005) both water intake and urine output over time.

**Figure 2:**
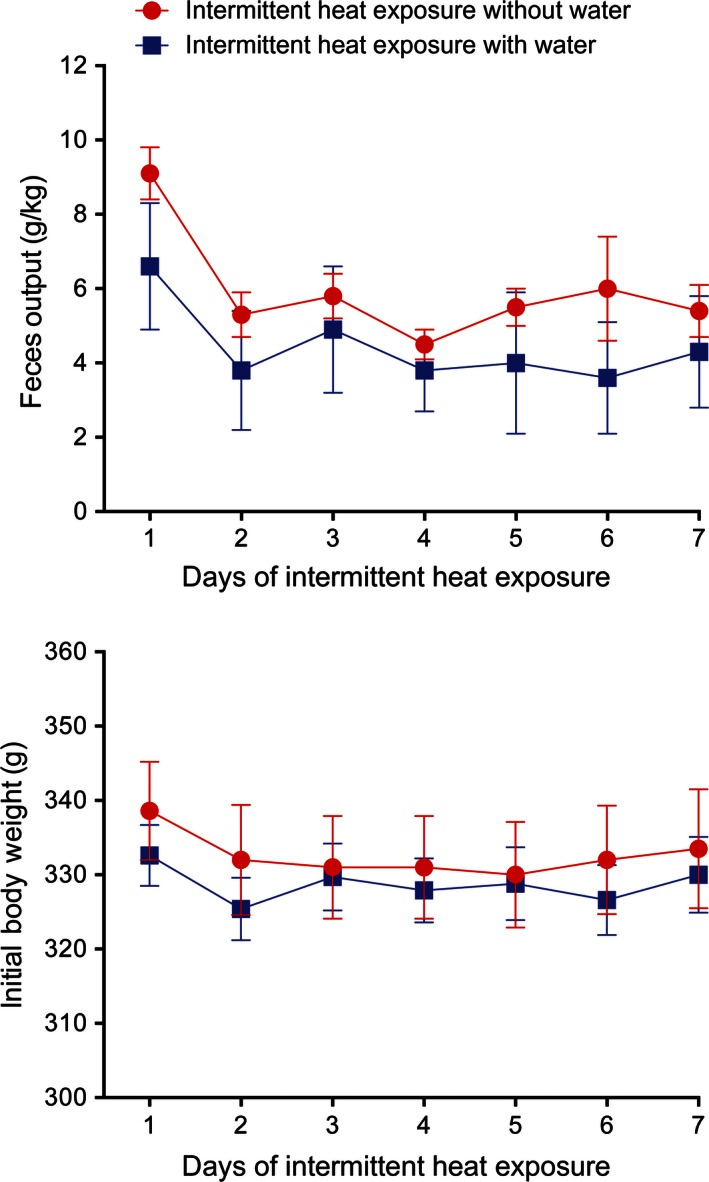
Mean ± SE feces output during (top) and body weight before (bottom) 4 h of exposure to 37.5°C for seven consecutive days with and without access to water. *N* = 6 per group. The decreases in both feces output and body weight over time were significant (*P* < 0.001).

In order to determine the effects of access to water on water balance during intermittent heat exposure, rats were exposed to heat for 4 h each day over the course of the study. Following 7 days of intermittent heat exposure, rats with and without access to water demonstrated reductions in evaporative water loss (*F*_6,60_ = 6.40, *P* < 0.005) (Fig. 1, top). The IH_w_ group had significantly (*F*_1,10_ = 11.28, *P* < 0.01) more evaporative water loss than the IH_w/o_ group but there was no significant interaction between group and time on evaporative water loss. The IH_w_ group demonstrated increases in both water intake (*F*_6,30_ = 5.05, *P* < 0.005) and urine output (Fig. 1, middle and bottom) with exposure day while the IH_w/o_ group demonstrated decreases in urine output over time. There were significant effects of group (*F*_1,10_ = 13.60, *P* < 0.005) and time (*F*_6,60_ = 5.38, *P* < 0.005) and a significant interaction between group and time (*F*_6,60_ = 7.98, *P* < 0.001) on urine output. Feces output during heat exposure (Fig. 2, top) decreased 42% on day 2 in both groups and remained stable for the rest of the experiment, with only a significant effect of time (*F*_6,60_ = 7.01, *P* < 0.001). Similarly, body weight before heat exposure (Fig. 2, bottom) was lower on day 2 then on day 1 in both groups and remained fairly stable for the rest of the experiment, with only a significant effect of time (*F*_6,60_ = 6.86, *P* < 0.001).

**Figure 3:**
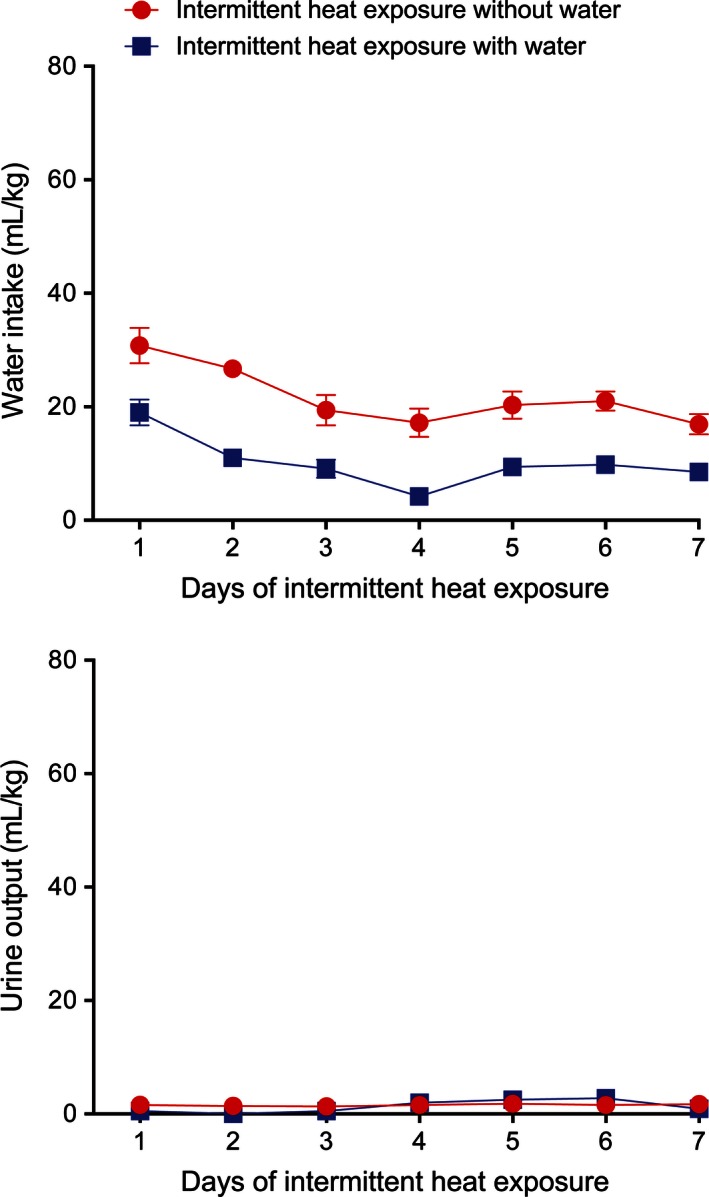
Mean ± SE recovery period water intake (top) and urine output (bottom) following 4 h of exposure to 37.5°C for seven consecutive days with and without access to water. *N* = 6 per group. During the recovery period, there was a significant decrease in water intake (*P* < 0.001) over time and the rats without access to water during heat exposure drank significantly *P* < 0.001) significantly more water than the rats with access to water during heat exposure. Urine output during the recovery period was significantly (*P* < 0.05) affected by days of heat exposure.

Each day following the 4 h heat exposure, all rats were moved to a 25 ± 0.5°C environmental chamber and allowed access to water. At all time points assessed, IH_w/o_ rats drank significantly (*F*_1,10_ = 130.03, *P* < 0.001) more water than the IH_w_ rats during this recovery period (Fig. 3, top). Water intake in both groups decreased initially (*F*_6,60_ = 12.72, *P* < 0.001) and then leveled off as the days of intermittent heat exposure continued. There was no significant interaction between group and time on postheat water intake. Urine output during the recovery period was low in both groups (Fig. 3, bottom) with only a significant effect of time (*F*_6,60_ = 2.97, *P* < 0.05).

**Figure 4:**
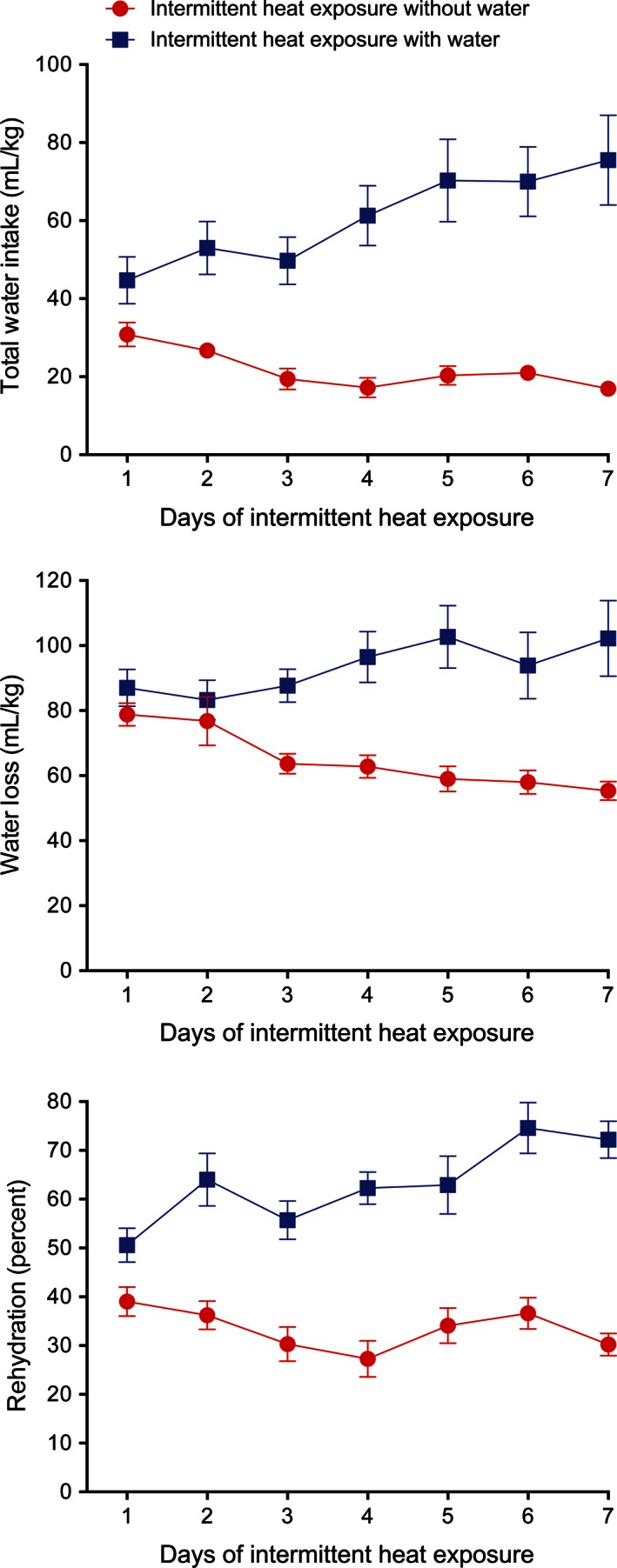
Mean ± SE total water intake (top), total water loss (middle), and percent rehydration (bottom) during and after 4 h of exposure to 37.5°C for seven consecutive days with and without access to water. *N* = 6 per group. There was a significant (*P* < 0.001) interaction between group and days of exposure on total water intake and water loss with the group having access to water during heat exposure having significantly (*P* < 0.001) higher intakes and losses. Percent rehydration was significantly (*P* < 0.001) higher in the rats that had access to water during heat exposure.

Total water intake and evaporative and urinary water loss measured over the course of the exposure period and the recovery period were analyzed. During the 7 days of intermittent heat exposure, the total water intake of the IH_w_ rats increased by 69%, while the total water intake of the IH_w/o_ rats decreased by 45% (Fig. 4, top). There was a significant effect of group (*F*_1,10_ = 37.53, *P* < 0.001) as well as a significant interaction between group and time (*F*_6,60_ = 5.84, *P* < 0.001). Water loss, which includes evaporative water loss and urine output during the exposure period and the 3‐hour urine output during the recovery period, increased with intermittent heat exposure for the IH_w_ group and decreased for the IH_w/o_ group (Fig. 4, middle) with a significant effect of group (*F*_1,10_ = 22.51, *P* < 0.001) and time (*F*_6,60_ = 6.19, *P* < 0.001) and a significant interaction between group and time (*F*_6,60_ = 4.42, *P* < 0.001). Of particular interest, percent rehydration (Fig. 4, bottom) was significantly (*F*_1,10_ = 52.84, *P* < 0.001) higher in the rats that had access to water during heat exposure than in the rats without access to water during heat exposure. Percent rehydration increased with successive heat exposures in the IH_w_ group but did not increase in the IH_w/o_ group. There was a significant effect of time (*F*_6,60_ = 4.80, *P* < 0.001) and a significant interaction between group and time (*F*_6,60_ = 6.14, *P* < 0.001) on percent rehydration. Taken together, these data demonstrate that the presence of water during 7 days of intermittent heat exposure gradually leads to increased water intake and water loss with an overall improvement in rehydration level.

### Long‐term intermittent heat exposure without access to water alters cellular and volemic thirst responses

**Figure 5:**
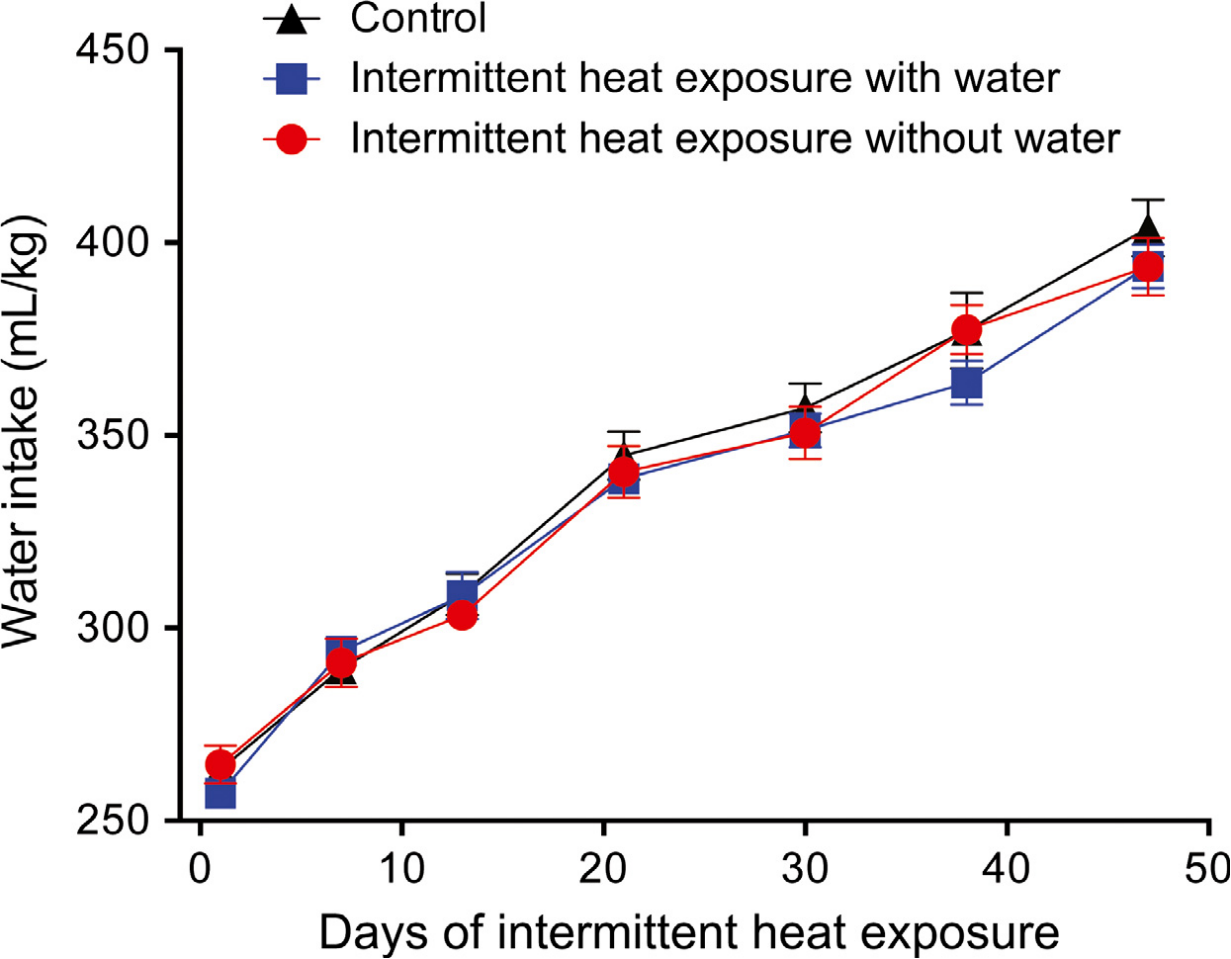
Mean ± SE body weight for the control, intermittent heat exposure with water, and intermittent heat exposure without water rats during the course of the study. There was a significant (*P* < 0.001) effect of time, but no significant effect of group or significant interaction between group and time on body weight.

**Figure 6:**
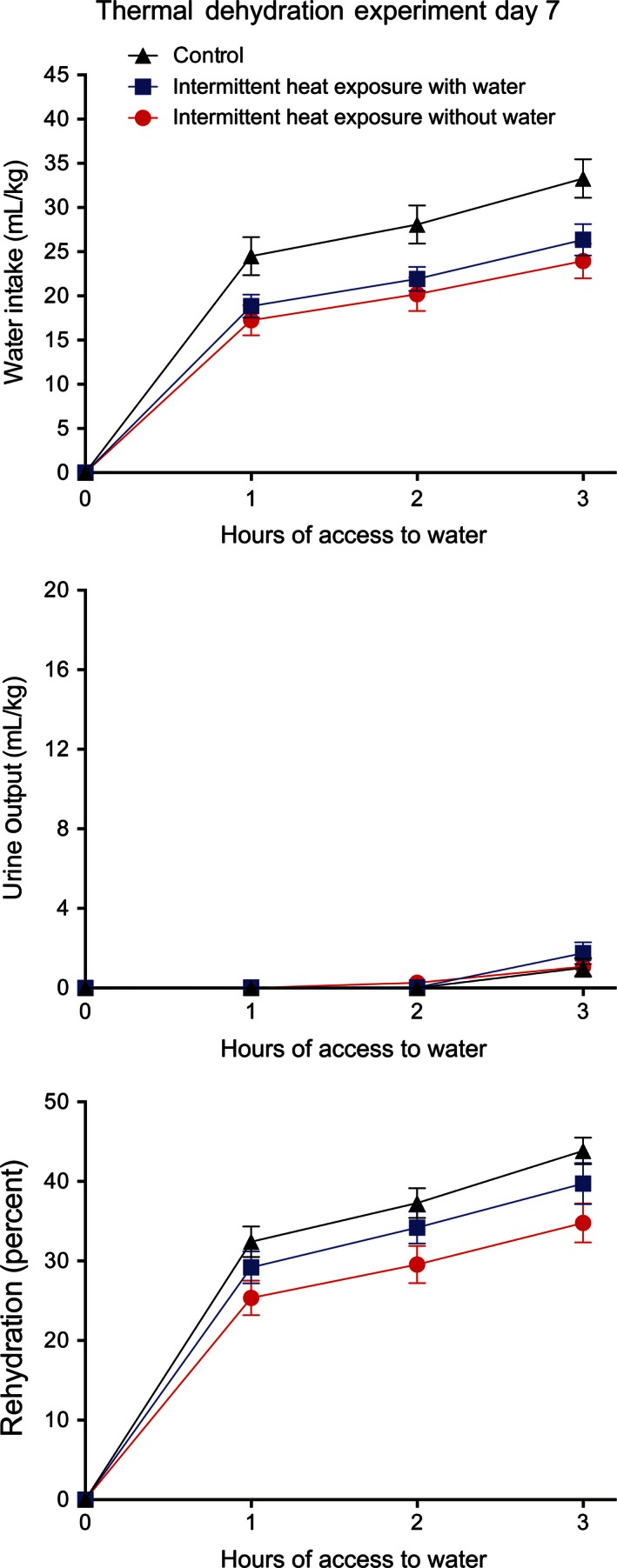
Mean ± SE water intake (top), urine output (middle), and percent rehydration (bottom) during 3 h of access to water after 4 h of exposure to 37.5°C without access to water for control rats and for rats exposed to 37.5°C for 4 h/day for 6 days with and without access to water. *N* = 12 per group. The control rats had significantly (*P* < 0.01) higher water intakes than did the other two groups and there was a significant (*P* < 0.05) difference among the three groups in percent rehydration with the control group having the highest levels.

To gain further insight into the effects of intermittent heat exposure in the presence or absence of water, thirst responses were assessed. The rats in each group gained weight throughout the experiment period (Fig. 5). One‐way ANOVA with repeated measures indicated a significant (*F*_5,33_ = 1255.15, *P* < 0.001) effect of time on body weight, but no significant effect of group or significant interaction between group and time. In the first experiment, performed on the 7th day of the study, thirst was induced by thermal dehydration. In the thermal dehydration experiment, the IH_w_ and IH_w/o_ rats had significantly (ANOVA *F*_2,33_ = 4.14, *P* < 0.05 and Tukey's tests *P* < 0.05) lower evaporative water losses (IH_w_ = 57.1 ± 2.4 mL/kg, IH_w/o_ = 59.2 ± 2.3 mL/kg) than the Con_w_ rats (66.7 ± 2.7 mL/kg). No significant differences occurred between urine output of any of the groups (Con_w_ rats = 7.6 ± 0.5 mL/kg, IH_w_ = 8.0 ± 0.7 mL/kg, IH_w/o_ = 8.7 ± 0.8 mL/kg) during heat exposure. IH_w_ and IH_w/o_ rats had significantly (*P* < 0.05 via Tukey's test following ANOVA *F*_2,33_ = 6.59, *P* < 0.01) lower feces output compared to Con_w_ rats during heat exposure (Con_w_ = 10.0 ± 0.9 g/kg, IH_w_ = 6.8 ± 1.1 g/kg, IH_w/o_ = 5.3 ± 0.7 g/kg). When allowed access to water during the recovery period, all three groups had high water intakes (Fig. 6, top) with significant effects of group (*F*_2,33_ = 5.44, *P* < 0.01) and time (*F*_2,66_ = 259.47, *P* < 0.001) but no significant interaction between group and time. All three groups also had low urine outputs (Fig. 6, middle) with only a significant effect of time (*F*_2,66_ = 186.23, *P* < 0.001). All of the groups rehydrated to less than 50% of water loss (Fig. 6, bottom) with the Con_w_ group rehydrating the most and IH_w/o_ group rehydrating the least. There were significant effects of group (*F*_2,33_ = 3.73, *P* < 0.05) and time (*F*_2,66_ = 212.95, *P* < 0.001) on percent rehydration, but no significant interaction between group and time.

**Figure 7:**
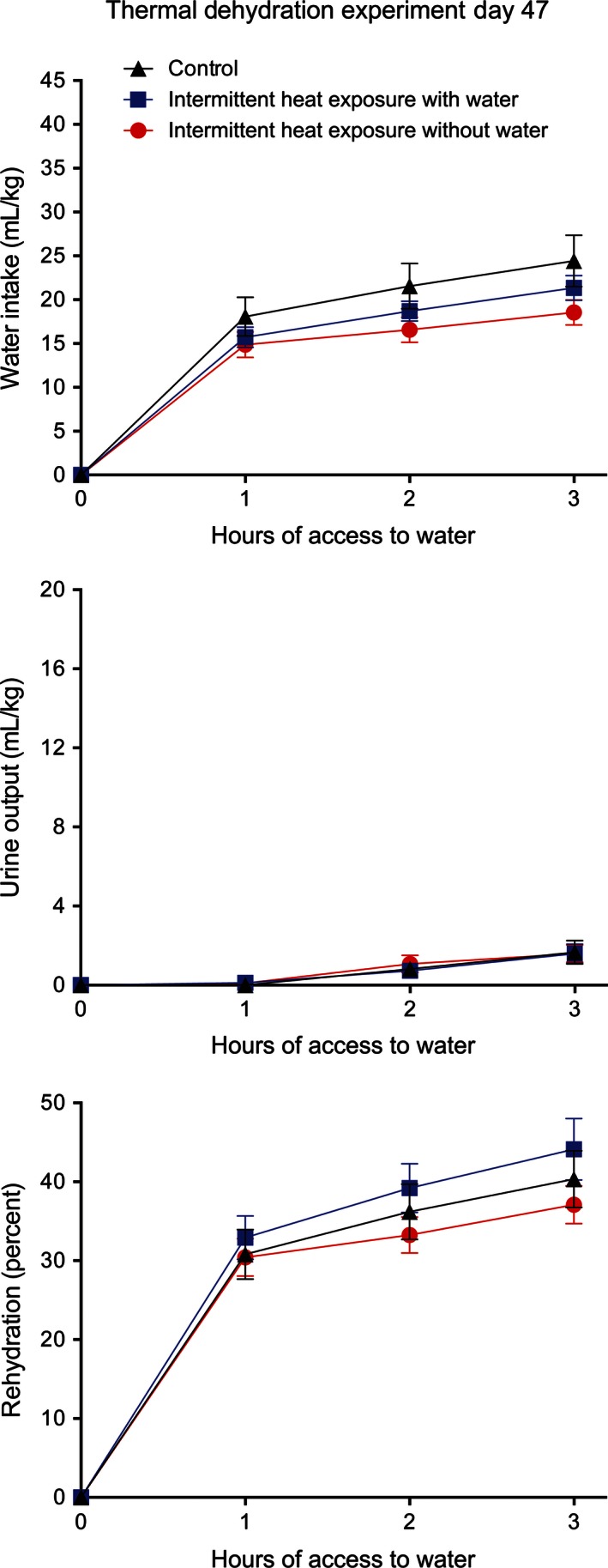
Mean ± SE water intake (top), urine output (middle), and percent rehydration (bottom) during 3 h of access to water after 4 h of exposure to 37.5°C without access to water for control rats and for rats exposed to 37.5°C for 4 h/day for 44 days with and without access to water. *N* = 12 per group. There was a significant (*P* < 0.02) interaction between group and time on water intake.

In order to determine if extended intermittent heat exposure had further effects on water balance responses to thermal dehydration, this experiment was repeated during the 47th day of the study and after 44 days of intermittent heat exposure. The evaporative water loss of the Con_w_ rats (51.0 ± 2.0 mL/kg) was again higher than the evaporative water loss of the IH_w_ rats (42.8 ± 2.6 mL/kg) and the IH_w/o_ rats (43.1 ± 2.3 mL/kg) with a significant (*F*_2,33_ = 3.96, *P* < 0.05) effect of group on evaporative water loss. The IH_w_ rats had a significantly (*P* < 0.05) lower evaporative water loss than the Con_w_ rats, while the evaporative water loss of the IH_w/o_ rats was almost significantly (*P* = 0.056) different from that of Con_w_ rats. Urine output (Con_w_ rats = 6.7 ± 0.8, IH_w_ rats = 5.8 ± 0.8 mL/kg, IH_w/o_ rats = 5.5 ± 0.8 mL/kg) was not significantly different among the three groups but feces output during exposure to the heat (IH_w_ = 2.6 ± 0.6 g/kg, IH_w/o_ = 2.4 ± 0.8 g/kg, Con_w_ = 8.2 ± 0.8 g/kg) was significantly (*F*_2,33_ = 19.12, *P* < 0.01) affected by prior heat exposure. Following three hours of access to water, the Con_w_ group had a higher water intake than the other two groups (Fig. 7, top). Although there was no significant effect of group alone on water intake, there was a significant (*F*_4,66_ = 3.25, *P* < 0.02) interaction between group and time as well as a significant (*F*_2,66_ = 133.95, *P* < 0.001) effect of time alone on water intake. Urine output (Fig. 7, middle) was low in all three groups with only a significant (*F*_2,66_ = 18.09, *P* < 0.001) effect of time. Rehydration (Fig. 7, bottom) after three hours of access to water varied from 37% to 44% with no significant differences among groups and only a significant effect (*F*_2,66_ = 93.75, *P* < 0.001) of time.

**Figure 8:**
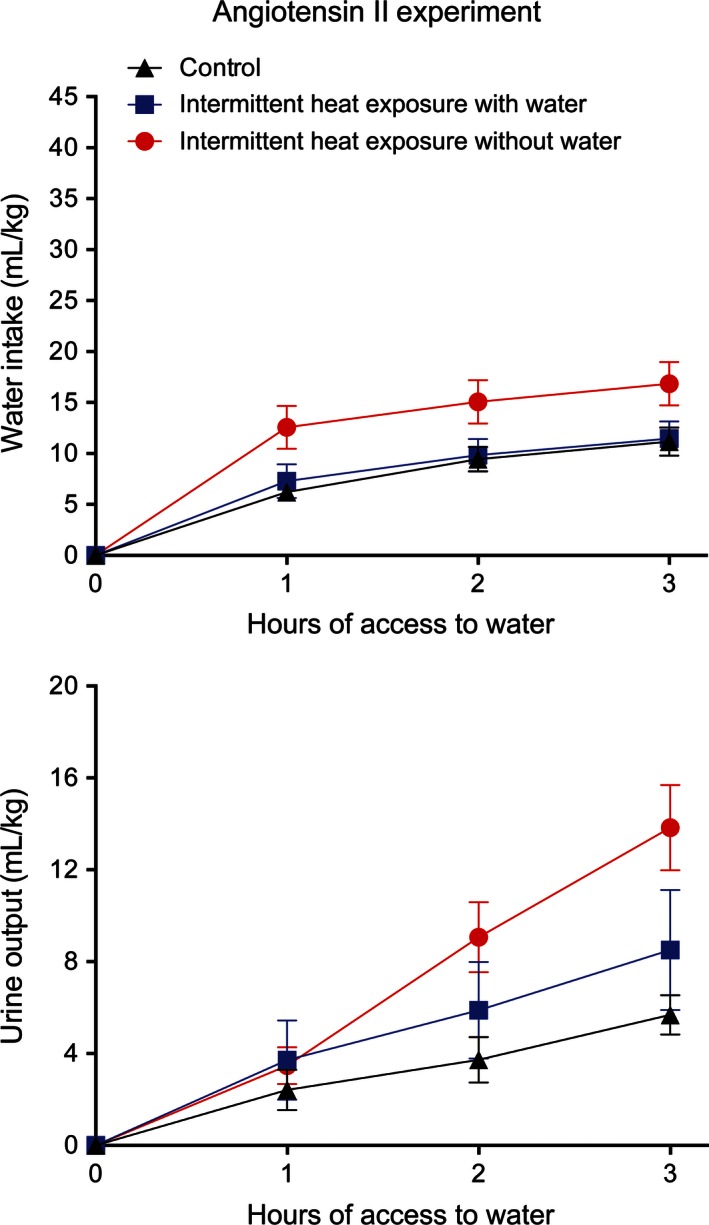
Mean ± SE water intake (top) and urine output (bottom) following administration of 200 *μ*g angiotensin II/kg for control rats and for rats exposed to 37.5°C for 4 h/day for 29 days with and without access to water. *N* = 6 per group. There was a significant (*P* < 0.05) effect of group on water intake and a significant (*P* < 0.001) interaction between group and time on urine output following administration of angiotensin II.

Although no differences were observed between IHw and IHw/o groups in response to thermal dehydration at day 47, differences were observed upon stimulation of the hypovolemic thirst pathway at day 30. The IH_w/o_ group had a higher water intake and urine output after administration of angiotensin II than the other two groups (Fig. 8). There were significant effects of group (*F*_2,15_ = 3.78, *P* < 0.05) and time (*F*_2,30_ = 193.83, *P* < 0.001) but no significant interaction between group and time on water intake. There was a significant effect of time (*F*_2,30_ = 57.17, *P* < 0.001) and a significant interaction between group and time (*F*_4,30_ = 7.17, *P* < 0.001) on urine output without a significant effect of group alone.

**Figure 9:**
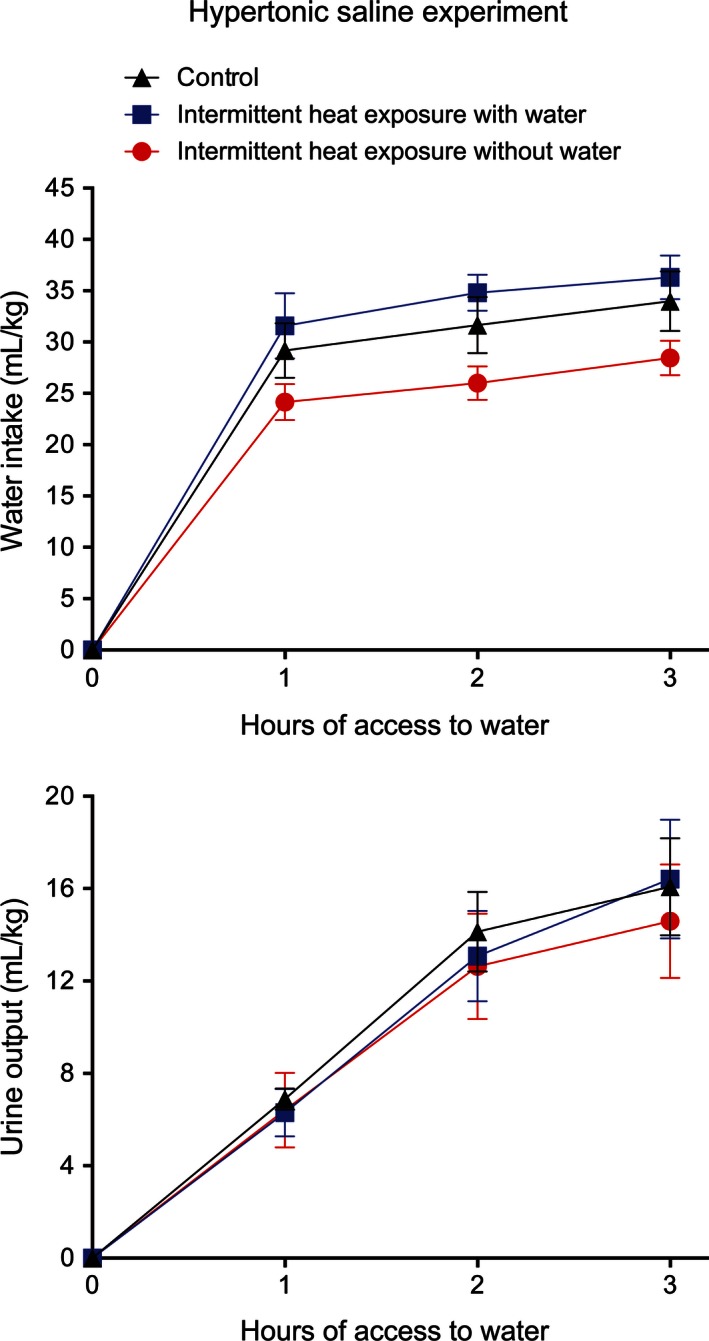
Mean ± SE water intake (top) and urine output (bottom) following administration of hypertonic saline for control rats and for rats exposed to 37.5°C for 4 h/day for 28 days with and without access to water. *N* = 6 per group. The effect of group on water intake neared significance (*P* = 0.055).

**Figure 10:**
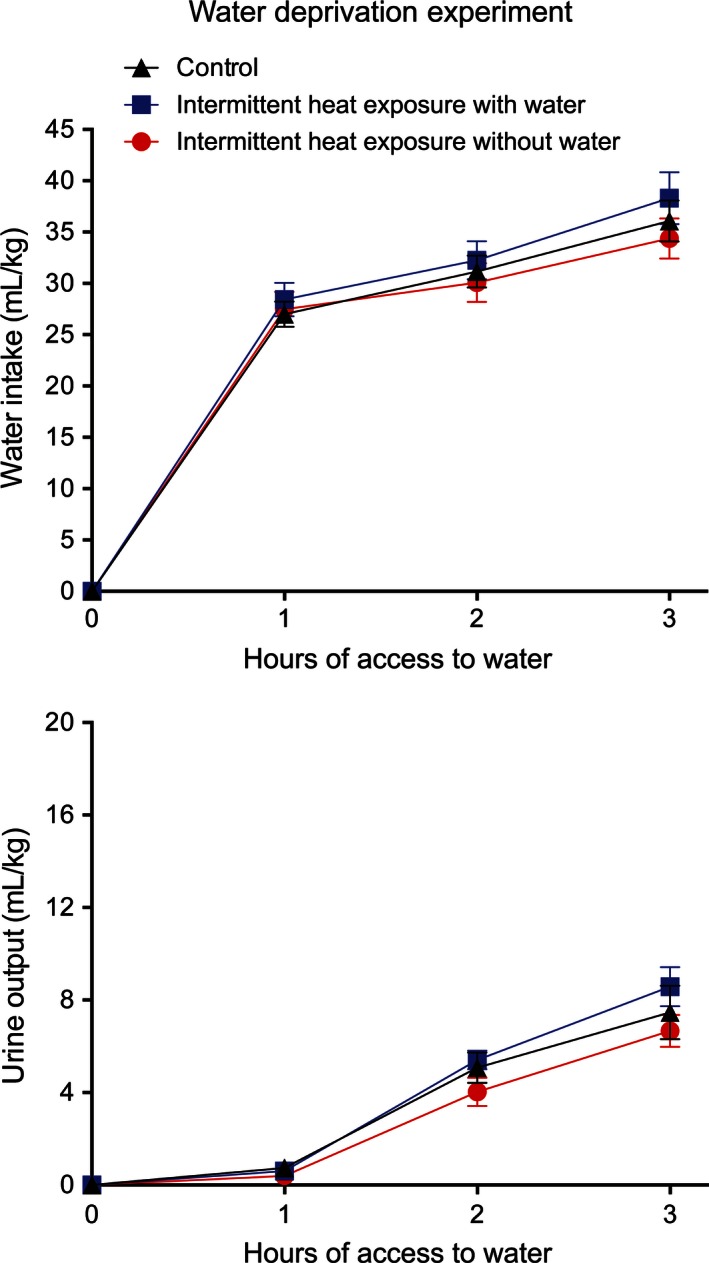
Mean ± SE water intake (top) and urine output (bottom) following 24 h of water deprivation for control rats and for rats exposed to 37.5°C for 4 h/day for 35 days with and without access to water. *N* = 12 per group. There were no significant effects of group on either water intake or urine output.

Differences between the IHw and IHw/o groups were also apparent upon stimulation of the cellular thirst pathway. Administration of hypertonic saline solution caused vigorous drinking and a high level of urine output in all three groups (Fig. 9). There were no significant differences among the groups in urine output with only time having a significant (*F*_2,30_ = 63.29, *P* < 0.001) effect. Water intake was lower in the IH_w/o_ rats than in the other two groups with the group effect not quite reaching statistical significance (*F*_2,15_ = 3.55, *P* = 0.055**)** and time having a significant (*F*_2,30_ = 96.14, *P* < 0.001) effect. There was no significant interaction between group and time for either water intake or urine output following administration of hypertonic saline. When allowed access to water following 24 h of water deprivation, water intake, and urine output did not differ among the three groups (Fig. 10), with only significant effects of time on water intake (*F*_2,66_ = 127.93, *P* < 0.001) and urine output (*F*_2,30_ = 178.00, *P* < 0.001).

## Discussion

The increased water loss for evaporative cooling during acute heat exposure in rats leads to thermal dehydration, which in turn leads to thirst and drinking when water is available (Hainsworth et al. 1968; Lund et al. 1969; Stricker and Hainsworth 1970; Grace and Stevenson 1971; Nose et al. 1985, 1986; Barney and West 1990). In previous studies on thermal dehydration‐induced thirst, water was made available either during (Hainsworth et al. 1968; Lund et al. 1969; Stricker and Hainsworth 1970; Grace and Stevenson 1971) or after (Nose et al. 1985, 1986; Barney and West 1990; Barney and Folkerts 1995; Whyte et al. 2004) heat exposure. In this study, we compared the effects of having water available during and after heat exposure to having water available only after heat exposure. Furthermore, we examined how intermittent heat exposure would affect water intake and urine output during thermal dehydration and other manipulations that increased water intake.

We found that intermittent heat exposure caused a progressive decline in heat‐induced evaporative water loss with reductions of 22% in the IH_w_ group and 28% in the IH_w/o_ group by the seventh day. Thus, a reduction in evaporative water loss similar to what is observed in rats heat acclimated by continuous heat exposure (Horowitz and Samueloff 1979; Horowitz et al. 1983; Barney et al. 2015) can be elicited using intermittent heat exposure. The reduction in activity and metabolic rate as well as the increased capacity for non‐evaporative heat loss that occurs with repeated heat exposure (Lewis et al. 1960; Shido and Nagasaka 1990a,b; Shido et al. 1991a,b) allow the rats to thermoregulate effectively with less use of evaporative heat loss and thus less thermal dehydration. The effects of intermittent heat exposure on core temperature were not determined in this study. However, Shido et al. (1989) reported that with repeated heat exposure, the rise in core temperature during heat exposure remained stable for the first 3 days of exposure and then increased over the next 7 days, although they used a lower exposure temperature than the one used in this study. It would be interesting to learn if repeated heat exposure that better activates evaporative water losses would lead to similar core temperature responses. As core temperature does not directly impact thermal dehydration‐induced thirst (Barney and Folkerts 1995), it is unlikely that any changes in core temperature responses that might occur with intermittent heat exposure would be responsible for the changes in water intake.

In this study, it was clearly demonstrated that access to water during heat exposure increased evaporative water loss, with the IH_w_ rats losing 57.4 mL/kg and the IH_w/o_ rats losing 46.4 mL/kg on the seventh day of intermittent heat exposure. These data indicate that evaporative water loss during heat exposure in rats is dependent on the state of hydration. This idea is supported by a prior study which showed that rats dehydrated by water deprivation prior to heat exposure had lower evaporative water losses than control rats at environmental temperatures of 32–38°C (Stricker and Hainsworth 1970). Interestingly, at environmental temperatures of 40–44°C, the dehydrated rats had similar evaporative water losses to control rats for the first half hour of exposure, but were unable to sustain the increase in water loss at later time points. Hydration and the ability to use evaporative cooling are critical for mitigating the deleterious effects of exposure to hot environments (Lund et al. 1969; Zurovski et al. 1991). Allowing rats access to water during heat exposure reduces body weight loss, hyperthermia, and the effects of heat exposure on plasma osmolality, plasma sodium, potassium concentrations, and hematocrit and increases heat tolerance time (Hainsworth et al. 1968; Grace and Stevenson 1971; Epstein et al. 1990; Zurovski et al. 1991). Taken together, these studies demonstrate that access to water during heat exposure significantly improves thermoregulatory ability.

Intermittent heat exposure with access to water leads to improved hydration through increased water intake during heat exposure. In this study, the amount of water ingested during the 4 h heat exposure period increased as the number of exposures increased. The IH_w_ rats drank 2.6 times more during the seventh heat exposure period than they did during the first heat exposure period. Although previous studies have demonstrated that rats allowed access to water during heat exposure drink substantial quantities of water (Hainsworth et al. 1968; Lund et al. 1969; Stricker and Hainsworth 1970; Grace and Stevenson 1971; Barney and Folkerts 1995), this study demonstrated an increase in water intake with repeated exposure. Increased water intake has also been reported for men who were heat acclimated by intermittent heat exposure with exercise (Greenleaf et al. 1967; Greenleaf et al. 1983; Edholm 1972). In contrast to studies on rats, in these studies evaporative water loss increased and the additional water intake was necessary to offset the increased water loss. Since the rats in this study had decreased evaporative water losses following intermittent heat exposure, the cause of their increased water intake is not clear. One possibility is that the rats become sensitized to the direct thermal effect of heat exposure on water intake (Lund et al. 1969; Grace and Stevenson 1971; Barney and Folkerts 1995). It is also possible that the reduced stress the rats exhibit with intermittent heat exposure starting on day 2 of exposure after higher levels on day 1, as indicated by reductions in activity (Lewis et al. 1960; Shido et al. 1991a) and feces output (Bonaz and Taché 1993; Morrow and Garrick 1997; Banji et al. 2014), allows the rats to be more attentive to water availability and to spend more time drinking.

Another possibility is that intermittent heat exposure reduces the responsiveness of the rats to presystemic or systemic signals that terminate drinking such as gastrointestinal distension or plasma sodium concentration (Blass and Hall 1976; Nose et al. 1985; Barney 1997; Stricker and Hoffmann 2007; Krause et al. 2010). Finally, and most likely, the increased water intake observed during intermittent heat exposure may be the result of learning (Holland 1991). Similar increases in water intake have been reported for schedule‐induced polydipsia (Lamas and Pellón 1995; DeCarolis et al. 2003) and for repeated episodes of water deprivation (Wotus and Engeland 2003). Any such learning appears to be specific to having access to water during heat exposure, as the rats that were repeatedly exposed to the heat without access to water did not show similar increases in water intake when water was provided postheat exposure. Further experiments are required to determine which, if any, of these possibilities explain the observed increase in water intake of the rats allowed access to water during intermittent heat exposure.

It should be noted that no measurements of sodium balance were carried out for these experiments nor were measurements continued beyond the 3‐hour recovery period each day. The loss of sodium in the saliva and urine during thermal dehydration is a major factor in limiting rehydration when only water is provided in the recovery period (Nose et al. 1985, 1986; Barney and West 1990; Barney et al. 1991; Barney 1997) and allowing rats access to sodium solution increases rehydration levels (Nose et al. 1985, 1986). Having access to food following the recovery period provided the rats with an opportunity to replace salt losses and thus continue the rehydration process. In this regard, measurements of sodium and water balance over a longer recovery period as were done by Nose et al. (1985, 1986) would be of value.

Intermittent heat exposure reduced water intake following heat exposure in both rats with and without access to water during heat exposure. For the IH_w/o_ rats, this reduction is most likely due to the decline in evaporative water loss and the magnitude of thermal dehydration. The lower water intakes of the IH_w_ rats reflects a further reduction in the level of thermal dehydration as these rats drank more water in the heat with intermittent heat exposure and lost less water by evaporation.

Recovery from thermal dehydration also depends on how well the kidney retains water. Surprisingly, since the rats were already losing considerable water for evaporative cooling, the IH_w_ rats lost a substantial amount of the ingested water in urine during the exposure period. In an earlier study, providing access to water during heat exposure prevented the increase in plasma arginine vasopressin (AVP) that accompanies thermal dehydration (Epstein et al. 1990). In this study, both groups of rats had low levels of urine output during the recovery period, similar to previous studies (Grace and Stevenson 1971; Barney and West 1990; Barney et al. 1995). It is not clear why the increase in urine output observed during exposure seen in the IH_w_ rats did not continue during the recovery period, since these rats were less dehydrated and presumably had lower AVP levels than the IH_w/o_ rats. Measurements of plasma AVP before, during, and after repeated heat exposure with and without access to water would be of interest in this regard.

The effect of intermittent heat exposure on water balance was very dependent on whether or not the rats had access to water only after or both during and after exposure as seen in the percent rehydration data. With intermittent heat exposure, the percent rehydration of the IH_w_ rats increased while the percent rehydration of the IH_w/o_ rats decreased. The incomplete hydration in the rats without access to water in the heat is due to the loss of the osmotic signal to drink following ingestion of water equal to about 50% of the water loss (Nose et al. 1985, 1986; Barney and West 1990; Barney 1997). The causes of the differences in percent rehydration between the IH_w_ and IH_w/o_ remain to be determined, but the results imply that having access to water during intermittent heat exposure may play a critical role in improving the ability of animals to recover from thermal dehydration.

The effects of having water to drink during intermittent heat exposure did not carry over when the IH_w_ and IH_w/o_ rats were exposed to the heat without access to water. Both IH_w_ and IH_w/o_ rats had reductions in evaporative water loss in the thermal dehydration experiments, and these reductions were similar after 1 and 7 weeks of intermittent heat exposure. Thus, the beneficial effects of intermittent heat exposure on evaporative water loss and thermal dehydration are maintained but do not increase further with extended repetitions. As with evaporative water loss, both IH_w_ and IH_w/o_ rats had reduced water intake following thermal dehydration. Intermittent heat exposure, with or without access to water, did not alter the renal response to thermal dehydration nor improve rehydration, even though a previous study indicated that plasma AVP concentration following thermal dehydration was increased by heat acclimation by continuous heat exposure (Epstein et al. 1990).

We also examined the effects of intermittent heat exposure on thirst induced by administration of hypertonic saline, which stimulates intracellular thirst, administration of angiotensin II, which mimics volemic thirst, and water deprivation, which stimulates both thirst pathways (Stricker and Sved 2000; Johnson 2007; Thornton 2010). We found that intermittent heat exposure with access to water had no effect on water intake induced by these three treatments. Intermittent heat exposure without access to water, however, reduced (although not quite significantly) water intake following administration of hypertonic saline and significantly increased water intake following administration of angiotensin II. These data suggest that intermittent heat exposure that leads to thermal dehydration may alter both intracellular and volemic thirst pathways, while intermittent heat exposure that does not lead to thermal dehydration because of access to water may not alter these pathways. Because water deprivation induces dehydration more slowly than heat exposure and activates both intracellular and volemic thirst pathways, it is not surprising that water deprivation did not alter water intake in the rats that did not have access to water during intermittent heat exposure as volemic thirst increased and intracellular thirst decreased in these rats.

## Summary

A better understanding of the effects of intermittent heat exposure on thirst and water balance is important as global temperatures and the risks of heat waves increase, particularly as factors, such as aging and chronic illness can contribute to the effects of heat stress. In this study, we found that intermittent heat exposure improved water balance in rats during acute heat exposure by reducing evaporative water loss. This reduction in evaporative water loss in turn leads to reductions in thermal dehydration‐induced thirst. The effects of intermittent heat exposure on water intake were dependent on whether the rats had access to water during the exposure periods. Rats with access to water during heat exposure increased water intake over days of exposure with an accompanying increase in urine output. The increased water intake appears to be a learned phenomenon and led to better rehydration than was observed in the rats that did not have access to water during heat exposure. Intermittent exposure to the heat reduced thermal dehydration‐induced thirst, an effect that was maintained over 7 weeks of exposure. Intermittent heat exposure did not alter water deprivation‐induced thirst. However, if the rats did not have access to water during intermittent heat exposure, intracellular thirst tended to be reduced and volemic thirst was increased. Further understanding of how water access during intermittent heat exposure affects acclimation to hot environments has the potential to lessen the risks of thermal dehydration.

## Conflict of Interest

None Declared.
